# Optimising planned medical education strategies to develop learners' person‐centredness: A realist review

**DOI:** 10.1111/medu.14707

**Published:** 2021-12-22

**Authors:** Aarti Bansal, Sarah Greenley, Caroline Mitchell, Sophie Park, Katie Shearn, Joanne Reeve

**Affiliations:** ^1^ Academy of Primary Care, Hull York Medical School University of Hull Hull UK; ^2^ Institute of Clinical and Applied Health Research University of Hull Hull UK; ^3^ Academic Unit of Medical Education Sam Fox House, Northern General Hospital Sheffield UK; ^4^ Research Department of Primary Care and Population Health University College London London UK; ^5^ Health and Wellbeing Research Institute – Postgraduate Research Centre Sheffield Hallam University Sheffield UK

## Abstract

**Context:**

Person‐centeredness is a stated aim for medical education; however, studies suggest this is not being achieved. There is a gap in our understanding of how, why and in what circumstances medical education interventions that aim to develop person‐centredness are successful.

**Methods:**

A realist review was conducted with a search of Medline, Embase, HMIC and ERIC databases and the grey literature using the terms ‘medical education’ and ‘person‐centred’ and related synonyms. Studies that involved a planned educational intervention in medical education with data on outcomes related to person‐centredness were included. The analysis focused on how and why different educational strategies interact with biomedical learner perspectives to trigger mechanisms that may or may not lead to a change in perspective towards person‐centredness.

**Results:**

Sixty‐one papers representing fifty‐three interventions were included in the final synthesis. Nine context–intervention–mechanism–outcome configuration (CIMOc) statements generated from the data synthesis make up our refined programme theory. Where educational interventions focused on communication skills learning or experiences without person‐centred theory, learners experienced dissonance with their biomedical perspective which they resolved by minimising the importance of the learning, resulting in perspective endurance. Where educational interventions applied person‐centred theory to meaningful experiences and included support for sense making, learners understood the relevance of person‐centeredness and felt able to process their responses to learning, resulting in perspective transformation towards person‐centredness.

**Conclusion:**

Our findings offer explanations as to why communication skills‐based interventions may be insufficient to develop learners' person‐centredness. Integrating experiential person‐centred learning with theory on why person‐centredness matters to clinical practice and enabling learners to make sense of their responses to learning, may support perspective transformation towards person‐centredness. Our findings offer programme and policymakers testable theory to inform the development of medical education strategies that aim to support person‐centredness.

## Abstract 2

By reviewing training strategies, the authors offer practical explanations for what does and doesn't work to support person‐centredness, arguing it is critical for us to explain why person‐centredness matters.

## 1 INTRODUCTION

Person‐centredness is recognised as an essential attribute for clinicians to deliver effective health care^1^ and is an espoused outcome for medical education.^2^ Person‐centred communication skills training is an established part of many medical education curricula^3^; however, there has been concern that person‐centred attitudes do not increase in learners as they progress through medical education and may decline.^4^, ^5^, ^6^, ^7^, ^8^, ^9^, ^10^ In recent years several studies have offered evidence on how certain types of medical education, such as longitudinal integrated clerkships (LICs),^11^, ^12^, ^13^ can support person‐centredness. However, we still have insufficient understanding as to how, why and in what circumstances the range of planned medical education programmes that aim to develop person‐centeredness are successful or not. This knowledge is important if we are to optimise medical education for the development of person‐centred practitioners.

Person‐centredness is based on an interpretivist epistemological framework^14^ which guides one's perspective on what constitutes health and one's clinical role. It is often described in contrast to a positivist biomedical perspective in which patients' reports of illness are taken to indicate the existence of disease processes and the clinician's ‘expert’ role is limited to accurate diagnosis and management of disease to cure or improve the patient's illness.^15^ A person‐centred perspective recognises that the person is a unique individual whose health is a complex interplay of biopsychosocial and other factors and whose health goals will be informed by their unique context and in terms of what matters to them.^16^, ^17^ It recognises patients' subjective knowledge and expertise and seeks to work with patients as equal partners in a shared decision making process.^16^ It also recognises that the quality of relationships influences health outcomes and that health care professionals' self‐awareness is a vital component of a person‐centred approach.^18^, ^19^ Mead and Bower's review of the empirical literature describe five conceptual domains of patient‐centredness: biopsychosocial perspective, ‘patient‐as‐person’, sharing power and responsibility, therapeutic alliance and ‘doctor‐as‐person.’^20^ Person‐centred theory involves the conceptual and evidential rationale for a person‐centred approach to clinical practice.^21^

The terms ‘patient‐centred’ and ‘person‐centred’ are often used interchangeably, and there is considerable overlap in the conceptual domains underpinning these terms. In recent years, there has been a move towards the term ‘person‐centred’ to acknowledge the personhood of an individual and not just their status as a patient.^22^ We therefore chose to use the term ‘person‐centred’ in our review.

Teaching of person‐centredness in medical education is often conceptualised in terms of skills and competencies.^23^ However, studies have shown that biomedical clinician perspectives may be a barrier to delivering person‐centred care^24^, ^25^ and it has been argued that a person‐centred approach requires a perspective shift from a focus on ‘what is the matter with you?’ to ‘what matters to you?’^26^ Various policy papers recommend that a workforce committed to person‐centred practice is an essential pillar in delivering person‐centred care^1^, ^27^ and that person‐centred values and principles are essential to guiding person‐centred behaviours.^28^ We queried whether there is a gap in medical education strategy and if the current focus on communication skills training may overlook person‐centred theory.

Our review sought to answer the following questions: What are the key mechanisms, triggered in particular contexts, that lead to the success or failure of planned medical educational interventions in developing person‐centredness? How might these explanations influence educational policy, practice, and research? The objective of our review was to produce testable theory useful to medical educators and policymakers. For this reason, we chose to focus on the planned education curriculum as opposed to the experienced curriculum which occurs in the wider health care context and over which medical education may have limited influence. We conceptualised the outcome of person‐centredness as a perspective and so chose to focus our review at the level of the learner.

## 2 METHODS

We chose to conduct a realist review as this is a practical methodological approach designed to inform policy and practice. Realist research is also particularly suitable for investigating complex interventions, such as those in medical education,^29^ and for synthesising qualitative, quantitative and mixed‐methods research. Unlike standard systematic reviews that seek to understand whether an intervention works, realist reviews have an explanatory focus and seek to understand what works for whom and in what circumstances.^30^ Our research focused on understanding outcomes at the level of perspective and so our use of the concept of ‘mechanism’ is at the level of human reasoning,^31^ where it represents learners' responses to the educational programme they are offered. This paper reports the review according to the Realist and Metanarrative Evidence Syntheses: Evolving Standards (RAMESES) publication standards.^32^ The review was conducted by a six‐person team which included four doctors with extensive experience in undergraduate and postgraduate medical education (AB, JR, SP and CM), a realist methodologist (KS) and an information specialist (SR).

### 2.1 Step (i): Developing an initial programme theory

Our first step was to describe an initial programme theory to guide data collection and analysis. We adopted three of the strategies outlined by Shearn et al.^33^: extracting tacit theories held by our research team, using concepts from substantive educational learning theory and extracting tacit theories from exploratory searches of the academic and grey literature. Details of our scoping searches are available at the open science framework (https://osf.io/qnkfh/?view_only=d1d36e578dd449a78ec62b13e2efebc5). These sources produced theory concepts that were configured to form an initial programme theory consisting of three draft programme theories based on the types of learning they represented: cognitive (theory of person‐centred care), constructivist (transformative learning environments) and experiential (clinical placements). The detail of our initial programme theory is in Appendix S1.

Our initial programme theory was checked with stakeholders planning, delivering and receiving undergraduate and postgraduate medical education. Their feedback confirmed that the initial programme theory provided a useful starting point for answering the research question. The stakeholder group discussed the scope of the review in terms of professional groupings and agreed to focus the review on medical students and doctors given that the diagnostic and management responsibilities of doctors are not shared with all health professionals and may have a specific impact in the development of a person‐centred approach. It was agreed that there were likely to be mechanisms in common across undergraduate and postgraduate learning and so papers from across the continuum of medical education should be included.

### 2.2 Step (ii): Searching for evidence

Our initial programme theory provided the framework to develop our systematic search strategy. Earlier scoping searches allowed us to refine our search strategy. For example, most papers found under the term ‘professionalism’ were concerned with the impact of the hidden (experienced but not planned) curriculum, whereas our review focus was on the planned curriculum. We searched multiple electronic databases (Medline, Embase and HMIC via the OVID interface and ERIC via the EBSCO interface on 15 July 2019) through Boolean combinations of free‐text and database‐specific subject heading terms for ‘medical education’ and ‘person‐centred’ and related synonyms. Full search strategies for each database can be found in Appendix S2.

We did not limit the search by including search terms on outcomes as we did not want to miss useful papers that may include data on person‐centred values, attitudes and beliefs without using these terms. In addition, relevant studies identified from a previous search for grey literature at the scoping stage were included. The search was limited to the English language and to results published since 2000 as we reasoned that this was when the concepts of patient‐centredness and person‐centredness became established in medical education. Additional papers relating to highly relevant studies were found using the CLUSTER method^34^ including citation tracking and contact with authors. As the synthesis progressed, a new element of theory emerged on ‘professionalism being understood as maintaining emotional distance’, and as is recommended in realist synthesis,^35^ we did additional searching, using Google Scholar, to look for evidence to support, refute or refine this element of theory.

### 2.3 Step (iii): Selection and appraisal of documents

Selection of articles was based primarily on the RAMESES identified principle of relevance, whether the article illuminated the research question and contributed to theory development.^35^ There was no restriction on the type of study eligible for inclusion. Screening was led by AB with a random sample of 10% of titles double appraised by JR, CM, SM, KS or SG at both abstract and full‐text stages. We included papers with a planned educational intervention for medical students and doctors with data on outcomes related to person‐centredness. In realist synthesis, the inclusion of relevant data from across disciplinary boundaries is encouraged to support theory development. We therefore agreed to include three papers that were returned in our search, which related to other health professionals as we felt they supported the overall development of programme theory. We excluded papers that were about the hidden curriculum, where there was no intervention or where the description of the intervention was too brief to derive explanation. Health care interventions that were specific to a particular policy context and not part of a formal medical educational programme, were also excluded (e.g. person‐centred medical home interventions in the USA). We also excluded papers that only reported outcomes at behavioural level (e.g. improvement in communication skills). Full inclusion and exclusion criteria can be found within the protocol published on the PROSPERO database (CRD42020168197).

### 2.4 Step (iv): Extracting and organising data

Before extracting the data, first read all the included full‐text papers to familiarise herself with the data. Data on study characteristics and summary findings for each paper was extracted into an Excel spreadsheet. In addition, each paper was assessed for rigour (high or low) based on whether its findings were considered coherent and plausible. Coding was both inductive, i.e., codes were identified from the data, and deductive, i.e., they were informed by the initial programme theory. Each paper was uploaded onto NVivo 12 (QSR International, Warrington, UK) qualitative data analysis software, to facilitate coding of text at a granular level. These coded data were grouped into concepts and then developed into ‘if–then’ explanatory statements. Explanations of each paper's findings were then transferred to the Excel spreadsheet for ease of looking at explanations across papers. These explanations were colour coded so we could easily see if they supported, refined or refuted the draft programme theories or whether they inspired new theory. A table with details of studies included in our realist synthesis is available in Appendix S3.

### 2.5 Step (v): Data synthesis

The data synthesis process involved several iterative cycles where we continually moved between data and theory to help us develop realist causal explanations for outcomes. The emerging findings were subject to critical appraisal at regular research team meetings to ensure the coherence and validity of the explanations. To develop our realist explanatory theories, we looked across papers to see if there were repeated patterns (demi‐regularities) between particular educational strategies and outcomes. We then looked for results and/or author suggestion and used abductive reasoning to see if we could explain how these outcomes occurred (mechanisms). Data synthesis then involved looking across the data for confirmatory and disconfirmatory evidence for our theories and, indeed, novel explanations that might explain the change in learner perspective or lack thereof.

A learner's perspective prior to learning was inferred from data indicating their response to the learning. We found that all interventional strategies that aimed to support person‐centredness validated the perspective of participants who leaned towards a person‐centred perspective. Learners who leaned towards a more biomedical perspective had variable responses to learning and this was the predominant learner perspective reflected in both our qualitative and quantitative data. At this stage, we chose to focus our review on the subset of data that provided explanations of the impact of educational strategies on learners who leaned towards a biomedical perspective. In line with RAMESES guidance,^35^ we checked with our stakeholders who agreed this focus would be most useful to them as educators and policymakers in terms of addressing the question of how planned medical educational strategies can be optimised to support learners' person‐centredness.

Several models are available for articulating theory configurations.^36^, ^37^ We found that separating the learner context from the educational strategy helped us to isolate the mechanisms leading to changes in perspective. We therefore chose to use the context–intervention–mechanism–outcome configuration (CIMOc) for our realist causal explanations. We grouped CIMOcs based on their outcome and working backwards were able to investigate the key features of the educational strategies that triggered the mechanisms leading to a change in person‐centred perspective (or not) and so to construct an overall programme theory.

## 3 RESULTS

**FIGURE 1:**
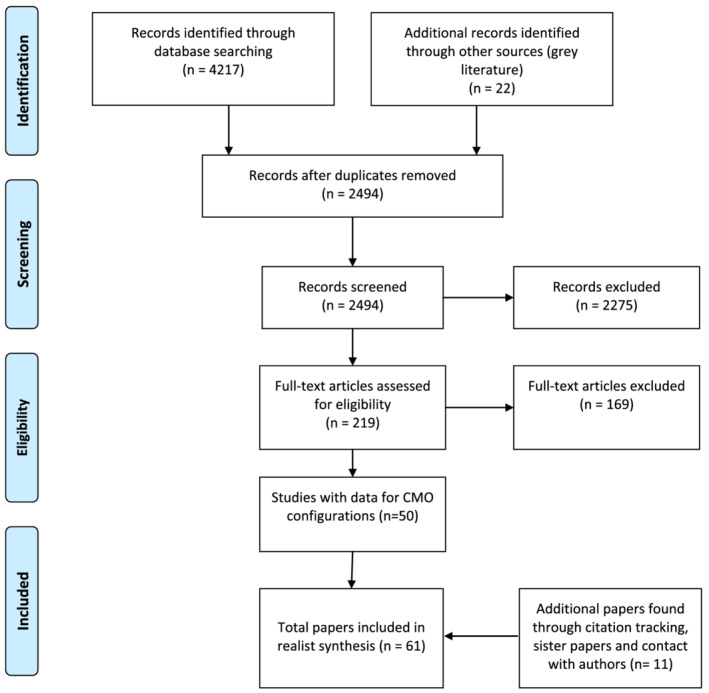
PRISMA flow diagram [Color figure can be viewed at wileyonlinelibrary.com]

The initial search yielded 4217 results. Figure 1 illustrates the filtering process that resulted in 61 papers or documents being included in the final realist synthesis. Eight papers or documents were looking at the same ‘intervention’, so the number of educational interventions looked at was 53. In 37 interventions, learners were undergraduates; in 11, they were postgraduate doctors‐in‐training; and in five, they were experienced clinicians or multidisciplinary teams in work. The educational interventions spanned the globe, although the majority were from North America and Europe (23 in North America, 20 in Europe, five in Asia, four in Australia and one in Africa).

There were three main types of interventional strategy: person‐centred communication skills training, patient experiences without person‐centred theory and interventions that offered various combinations of person‐centred theory, engagement with patient narrative or real patients over time (meaningful experience) and opportunities to make sense of responses to learning. The length of interventions varied widely from brief single sessions to regular learning in small groups over 4 years. Thirty‐seven papers offered qualitative data, 14 had quantitative data, eight papers had both qualitative and quantitative data and two were descriptions of interventions (sister papers) without data.

### 3.1 Refined programme theory

**FIGURE 2:**
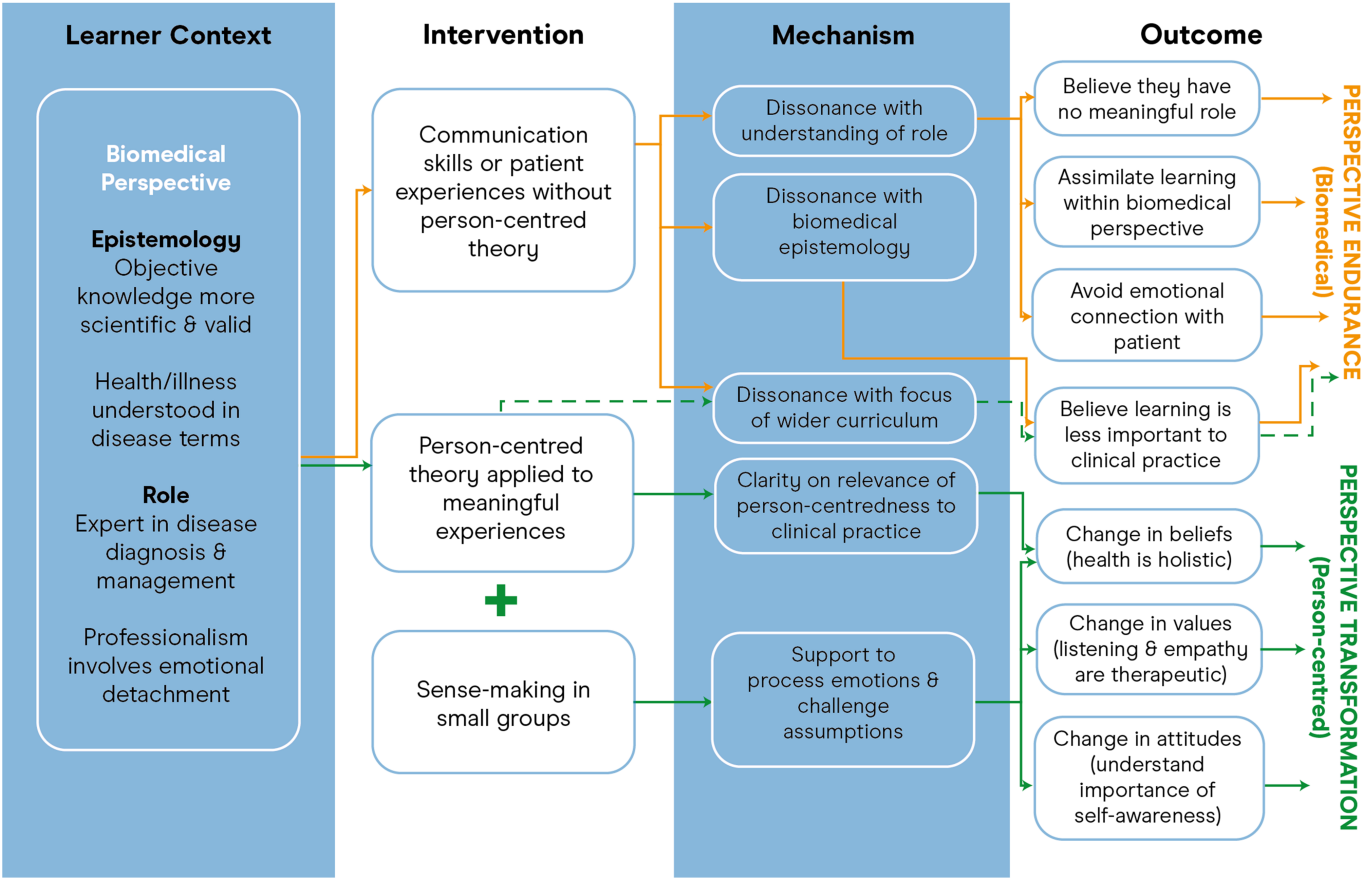
Refined programme theory [Color figure can be viewed at wileyonlinelibrary.com]

Our refined programme theory consists of nine CIMOcs that explain how educational interventions that aim to develop person‐centredness may or may not succeed (Table 1). Our analysis found that these explanations applied to leaners across the medical education continuum (undergraduate, postgraduate and continuing professional development). Figure 2 is a visual representation of our refined programme theory.

**TABLE 1:** Nine CIMOcs explanatory statements from data synthesis

Perspective endurance: How educational interventions may fail to develop person‐centredness
Mechanism 1: Dissonance with role
CIMOc 1: If learners with a biomedical perspective (C) participate in experiences with patients who are not acutely unwell or where there is no cure (I), then this may cause dissonance with learners' understanding of their role in terms of disease diagnosis and management (M) which may be resolved by learners believing they have no meaningful role with these patients (O)
CIMOc 2: If learners with a biomedical perspective (C) participate in communication skills teaching without person‐centred theory (I), then this may lead to dissonance with learners' understanding of their role in terms of disease diagnosis and management (M) which may be resolved by learners assimilating learning within their biomedical perspective (O)
CIMOc 3: If learners with a biomedical perspective (C) participate in learning which involves connecting with patients' emotions (I), then this may lead to dissonance with learners' understanding of professionalism as emotionally detached from patients (M) which may be resolved by learners avoiding connecting with patients' emotions to maintain professional norms and protect themselves from distress (O)
Mechanism 2: Dissonance with epistemology
CIMOc 4: If learners with a biomedical perspective (C) participate in a person‐centred learning experience without person‐centred theory, (I) then this may create dissonance with learners’ understanding of valid knowledge and the concepts of ‘health’ and ‘illness’ in disease terms (M) which may be resolved by learners believing the learning is less important for future practice (O)
Mechanism 3: Dissonance with focus of wider curriculum
CIMOc 5: If learners with a biomedical perspective (C) participate in a person‐centred learning experience that does not integrate with the wider curriculum (I), then this may lead to dissonance with the perceived biomedical focus of the wider curriculum (I) which may be resolved by learners minimising the importance of the learning for future practice (O)

Perspective transformation: how educational interventions may succeed in developing person‐centredness
Mechanism 1: Clarity on relevance of person‐centredness to clinical practice
CIMOc 6: If learners with a biomedical perspective (C) participate in learning which applies person‐centred theory to meaningful experiences (I), then this may result in greater clarity on the relevance of person‐centredness to clinical practice (M) which may result in learners understanding health as a holistic construct and valuing their role in facilitating personalised health care (O)
Mechanism 2: Support to process emotions and reflect critically on assumptions
CIMOc 7: If learners with a biomedical perspective (C) are provided with the opportunity to make sense of their responses to learning in supportive small groups with relational continuity (I), then the experience of being listened to non‐judgementally and feeling able to process their emotional responses (M) may result in learners valuing listening and empathy as therapeutic (O)
CIMOc 8: If learners with a biomedical perspective (C) are provided with the opportunity to make sense of their responses to learning in supportive small groups with relational continuity (I), then reflecting critically on the diversity of the groups' experiences and perspectives (M) may result in learners recognising health as a holistic concept and valuing their role in facilitating personalised health care (O)
CIMOc 9: If learners with a biomedical perspective (C) are provided with the opportunity to make sense of their responses to learning in supportive small groups with relational continuity (I), then reflecting critically on their assumptions in relation to patient care (M) may result in learners becoming more self‐aware and understanding the importance of self‐awareness to person‐centred care (O)

Abbreviation: CIMOc, context–intervention–mechanism–outcome configuration.

### 3.2 Perspective endurance: How educational interventions may fail to develop person‐centredness

Five explanatory statements (CIMOcs 1–5) explain how educational interventions that aim to increase person‐centredness may fail to do so. The mechanisms triggered in learners that led to perspective endurance all relate to cognitive dissonance: the discomfort and uncertainty that results when beliefs, values or attitudes are not consistent with each other.^38^ Our analysis described three types of dissonance related to role, epistemology and the perceived biomedical focus of the wider curriculum.

#### 3.2.1 Mechanism 1: Dissonance with understanding of role

Learners' understanding of their role as a doctor included the purpose of the role and what was considered professional behaviour. Most interventions that contributed to dissonance with role involved person‐centred communication skills training.^39^, ^40^, ^41^, ^42^, ^43^, ^44^, ^45^, ^46^, ^47^, ^48^, ^49^ A few interventions involved experiences with patients that were relatively brief but did not offer meaningful connection or continuity with patients or any person‐centred theory.^50^, ^51^, ^52^ Analysis revealed that dissonance in this understanding of role led to three outcomes.

##### CIMOc 1: Learners believe they have no meaningful role

Where learners believed that the main role of the doctor is to help through biomedical disease diagnosis and management, and the learning intervention involved patients who were not acutely unwell,^49^, ^50^, ^53^, ^54^ some struggled to see the relevance of this learning to their future role.

One commented that the requirement to follow a patient, who was relatively well, was a ‘dumb idea’, adding, ‘I did enough volunteering during undergraduate’.^53^

On the other hand, if the patient was suffering but there was no cure (such as in end‐of‐life situations), their disease‐based understanding of role could lead them to feel powerless to help.^45^, ^46^, ^55^

The situation is extremely difficult, and I would probably feel inadequate, wanting to help the patient but having no means to do that …^46^

In both situations, the feeling that they had no meaningful role with patients whose needs did not fit with an expertise in disease diagnosis or management, resulted in perspective endurance.

##### CIMOc 2: Learners assimilate learning within their biomedical perspective

Within the subset of papers where the intervention involved communication skills training,^39^, ^40^, ^41^, ^42^, ^43^, ^44^, ^45^, ^46^, ^47^, ^48^, ^49^, ^56^ some learners dealt with the role dissonance they experienced by incorporating person‐centred approaches into their existing biomedical understanding of role.^39^, ^40^, ^42^, ^44^, ^47^, ^48^ These learners understood the purpose of person‐centred approaches as increasing their ability to be effective in their biomedical role. For instance, the purpose of listening was understood to get better information for diagnosis or to create the trust required for patients to comply with their advice.

I think it's important to have a good relationship with your patients because they are more likely to do what you say …^49^

Papers that observed learners' consultations noted that strategies such as listening were used instrumentally. Listening did not demonstrate genuine curiosity for the patient and what mattered to them, as cues were left unattended.

At the end of the curriculum the students in our study were inviting the patient openly and they were listening … The students often omitted patients' life experiences and did not respond to their emotions.^48^

##### CIMOc 3: Learners avoid emotional connection with patients to maintain professional norms and protect self from emotional distress

Learners' qualitative comments revealed that they understood professional behaviour to involve emotional detachment from patients in order to remain clinically objective. Where learners' experienced learning that involved connecting with people who were suffering, this understanding of their professional role led to uncertainty on how to respond. Learners experienced a conflict between responding as a human being or as a professional, as well as uncertainty on how to manage the distress that they felt. Learners resolved their dissonance and uncertainty by avoiding emotional connection with patients to maintain what they understood to be professional norms and also to protect themselves from emotional distress. This avoidance of emotional connection was both observed in learner behaviour^45^, ^48^ as well as discussed by learners in response to their learning.^43^, ^46^, ^52^, ^57^

One student wrote about her meeting with a married couple where one of them was dying. The spouse started to cry when they approached the topic of death. The student wanted to comfort her but refrained: I wish I'd put my hand on the woman's shoulder because that would have been human, even though it would not have been professional.^52^

If I become too involved, my own life becomes too distressing. The most difficult thing for me … may be keeping up ‘the professional role’, not getting too involved in the patient's feelings.^46^

#### 3.2.2 Mechanism 2: Dissonance with epistemology

##### CIMOc 4: Learners believe learning is less important to future clinical practice

Many papers revealed that learners had a positivist view of knowledge and considered valid knowledge to be composed of ‘hard’ objective facts. Knowledge that required interpretation or was based on individual experience was considered less scientific and less valid as illustrated by how it referred to as ‘soft’, ‘touchy‐feely’ or ‘fluffy’.^40^, ^42^, ^58^

The ways in which Sundown Medical School students opposed the ‘scientific’ to the ‘human’ side of medicine, discussed their ethics teaching, and related to the subject of mental illness as difficult, threatening and less concretely scientific all appeared to be indicative of understandings of knowledge that were quite strongly polarised between ‘hard’ and ‘soft’ forms …^42^

Learners experienced confusion as to how to integrate knowledge from subjective patent experience and wider health determinants with disease‐based clinical practice. This dissonance between positivist epistemological understandings of knowledge and learning, which was subjective and interpretive in nature, was resolved by believing such learning was less important to their future practice.

For these medical students and (maybe some or even) many medical scientists, the discursive nature of social science often makes it seem less certain, more contingent than natural scientific ‘fact’ and therefore less valid.^51^

A lack of knowledge of person‐centred theory, such as the rationale for a bio‐psycho‐social formulation of health, was identified as a key reason that learners struggled to engage with person‐centred learning.

Many students also reported scruples about exploring the patient's life circumstances, in particular, social relations and working conditions. The students revealed a lack of knowledge about the relevance of psychosocial factors to health and disease. Thus, they perceived a holistic perspective as unnatural.^44^

Papers with quantitative data that compared cohorts with different interventions showed a decline in person‐centred attitudes in cohorts where person‐centred theory was not clearly integrated into learning.^59^, ^60^ The data supporting this CIMOc were found across undergraduate and postgraduate settings and from all geographical areas.^41^, ^42^, ^44^, ^51^, ^53^, ^56^, ^61^, ^62^

#### 3.2.3 Mechanism 3: Dissonance with focus of the wider curriculum

##### CIMOc 5: Learners minimise importance of learning for future clinical practice

Many learners took their cue about the relative importance of learning based on whether it was part of the core curriculum and the relative weight given to disease‐based learning in the curriculum. This led learners to continue to separate knowledge into hierarchical categories, believing that person‐centeredness was not as important as ‘scientific’ learning or as important to future practice.^42^, ^43^, ^54^, ^55^, ^61^, ^63^

Lack of integration with the focus of the wider curriculum affected all types of interventions, as shown by the broken green line in Figure 2. Some educational interventions were integrated as a thread running through the curriculum across two or more years^39^, ^60^, ^64^, ^65^; however, none of the educational interventions were perceived as fully integrated with the focus of the wider curriculum in terms of what students were taught, what they experienced during clinical placements or what was assessed.

Another student wanted to be both academically skilled and empathetic but felt that being bio‐medically skillful is given higher priority throughout medical school. In his opinion, one learns only to diagnose, refer patients, and relate to a time schedule during medical school.^43^

### 3.3 Perspective transformation: How educational interventions may succeed in developing person‐centredness

Four explanatory statements from our data synthesis (CIMOCs 6–9) explain how educational interventions may lead to perspective transformation towards person‐centredness (Table 1). Critical analysis of these four statements identified two overarching mechanisms that prompted change towards person‐centredness: greater clarity on the relevance of person‐centredness to clinical practice and support to process emotions and challenge assumptions. Thirty‐six papers contributed to explanations on perspective transformation towards person‐centredness.^49^, ^58^, ^59^, ^63^, ^64^, ^65^, ^66^, ^67^, ^68^, ^69^, ^70^, ^71^, ^72^, ^73^, ^74^, ^75^, ^76^, ^77^, ^78^, ^79^, ^80^, ^81^, ^82^, ^83^, ^84^, ^85^, ^86^, ^87^, ^88^, ^89^, ^90^, ^91^, ^92^, ^93^, ^94^, ^95^ Out of these papers, 31 interventions offered a combination of the components that enabled both mechanisms to be triggered: person‐centred theory, meaningful experiences and opportunity to make sense of one's responses to learning. Only five papers that contributed to these explanations offered experiences without theory or sense‐making opportunities.^63^, ^68^, ^73^, ^84^, ^87^ The experiences offered in these five interventions were either longitudinal in nature or involved an active person‐centred role in caring for patients, both of which are likely to have enabled students to personally reflect on the relevance of person‐centredness to clinical practice.

#### 3.3.1 Mechanism 1: Clarity on relevance of person‐centredness to clinical practice

##### CIMOc 6: Understand health as holistic and value personalisation of care

Interventions that applied person‐centred theory to meaningful experiences helped learners understand the relevance of a person‐centred approach to patient care.^53^, ^58^, ^59^, ^60^, ^65^, ^66^, ^69^, ^71^, ^72^, ^74^, ^75^, ^77^, ^78^, ^82^, ^83^, ^86^, ^87^, ^89^, ^90^, ^94^, ^96^ The contribution of theory to these interventions varied from didactic introductions on the concepts and rationale for a person‐centred approach^60^, ^71^, ^72^, ^74^, ^82^, ^94^ to regular discursive and interactive engagement with theory and application.^58^, ^65^, ^70^, ^77^, ^85^, ^89^ Interventions with brief theoretical framing could support significant changes to perspective if the rest of the intervention supported the application of this theory to practice through discussion or through experience.^60^, ^75^, ^82^, ^94^

For undergraduate students, experiences that enabled them to engage with patients over time^63^, ^65^, ^71^, ^73^, ^76^, ^84^ and/or take an active role in patient care,^74^, ^83^ were more compelling and memorable.

In the cardiology ward we debated about the cause of her latest complaint of chest pain, new infarct or psychological and then rush on to the next patient when she refused angiogram again. On hindsight, the aetiology may not be relevant as she does not want any angiogram or intervention and she is on maximum doses of every cardiac medication. As a guest in her home, I sit beside her rather than stand over her, and I am forced not to rush. I realise she is a person and not a bed number …^84^

#### 3.3.2 Mechanism 2: Support to process emotions and reflect critically on assumptions

Learning experiences that integrated theory with meaningful experiences also generated powerful emotional responses and challenged learner assumptions.

Students remarked that despite their understanding of the pathophysiology of diabetes and its complications, they ‘had no appreciation how it really impacts someone on a daily basis and how they really have to fit it into all their daily activities’. The students described themselves as ‘being floored’ by their discussion.^65^

The interventions with the clearest qualitative data on perspective transformation towards person‐centredness all offered opportunities for learners to make sense of their responses to their learning.^49^, ^53^, ^58^, ^59^, ^60^, ^66^, ^67^, ^70^, ^72^, ^76^, ^78^, ^80^, ^82^, ^88^, ^89^, ^90^, ^91^, ^92^, ^95^, ^96^, ^97^, ^98^ Facilitated small group environments where there was relational continuity of peers and facilitator enabled the establishment of belonging and trust most likely to trigger this mechanism. Across papers where learners were offered regular small group learning, they expressed the importance of a ‘safe’ and ‘non‐judgemental’ setting to enable them to express their feelings and reflect critically on their thoughts and assumptions.^49^, ^53^, ^58^, ^70^, ^77^, ^80^, ^92^, ^96^, ^98^

##### CIMOc 7: Value listening and empathy as therapeutic

Educational interventions that offered leaners the opportunity to debrief on their emotional responses in a non‐judgemental environment where they felt safe, enabled learners to experience first‐hand the therapeutic power of listening and being listened to.^53^, ^58^, ^65^, ^70^, ^80^, ^86^, ^91^, ^92^, ^98^ This enabled learners to see both the holistic nature of health and their own potential contribution to patient health through listening and empathy. This change in perspective helped learners feel able to help patients in situations where they would have previously felt they had no role.

I will spend extra time with my patients if they need it, but I felt in some ways that it was kind of sucking me dry … I would feel frustrated, like what else can I do? … but [now] I feel OK just to listen and be present with them … and I think that in some ways that helps them more … and that is a wonderful thing that you can do for patients … I just needed to learn that myself, I guess.^91^

Additionally, the sharing of emotional responses in small groups helped learners see that emotional responses were common and helped validate and normalise their feelings.^53^, ^58^, ^65^, ^70^, ^76^, ^80^, ^86^, ^91^, ^92^, ^98^ Having been treated as individuals who mattered, they felt more committed to taking this approach into their professional practice.

It has changed my attitude in the sense of knowing that there are people who care about my wellbeing as a student. And because I have received, I also want to give back.^98^

##### CIMOc 8: Understand health as holistic and value personalisation of care

Small groups coming together to reflect on their experiences with patients were able to recognise how patients with similar conditions may have completely different experiences of their illness and that this did not necessarily relate to the severity of the disease. The small group setting illustrated to learners how their own responses, attitudes, assumptions and perspectives varied in response to patient stories. Not only did this diversity of experience help learners benefit from the entire group's experience, but it also demonstrated how personal circumstances, perspectives and values matter in clinical decision making.^53^, ^58^, ^65^, ^70^, ^86^, ^88^, ^92^

Another student, influenced by the diversity of the experiences with diabetes she had learned about in small group stated, ‘I came to the realization that people are all really different that have this same disease’, while another student concluded, ‘it's just kind of a matter of getting to know them and finding out what's important to them’.^65^

##### CIMOc 9: Understand the importance of self‐awareness

Meaningful interactions with patients led learners to recognise how often their assumptions were misplaced. The opportunity to question these assumptions in small groups and to reflect critically on the norms, values and beliefs underpinning them, helped learners recognise the impact that assumptions have on clinical decision making and patient care. This in turn fostered a recognition of the importance of self‐awareness to practicing good clinical care.^58^, ^65^, ^66^, ^70^, ^85^, ^86^, ^91^, ^92^, ^95^, ^98^, ^99^ The recognition of one's own fallibility and humanity helped learners feel more open to connecting with patients from a position of humility, respect and curiosity.

Each month, faculty and attendings working with the house staff meet to debrief the team about their experiences. The most striking and consistent observation is how often house staff report ‘being surprised’ by what they have learned about their patients. This deeper insight, in turn, has repeatedly led to opportunities to provide better patient care.^85^

Some interventions in small groups specifically included specific self‐awareness training skills, such as mindfulness, and these helped learners be more aware of their emotions and thoughts as well as giving them strategies for addressing them.^91^, ^92^, ^95^, ^98^

This course has been about self‐awareness for me. I have learned to better recognize what is going on for me physically and emotionally. I have also learned a new set of tools for dealing with the stresses in life.^98^

## 4 DISCUSSION

### 4.1 Summary of findings

Our realist review set out to answer how, why and in what circumstances planned medical education programmes that aim to develop person‐centredness are successful or not. We located this review at the level of individual learner, conceptualised person‐centredness as a perspective and focused our review on the responses of learners who leaned towards a biomedical perspective. It has been widely observed that medical education has found it challenging to develop person‐centredness in health care practitioners.^4^, ^5^, ^6^, ^7^, ^8^, ^9^, ^10^ The explanations in our review deepen our understanding of this problem and offer new solutions for future practice. Our refined programme theory found two main interventional strategies that can help learners towards person‐centredness: person‐centred theory applied to meaningful experiences and small group environments that support learners to do the emotional work and critical reflection needed to challenge existing meaning frameworks. We discuss these strategies in relation to the wider literature.

### 4.2 Person‐centred theory applied to meaningful experiences

Our synthesis found a key role for applied person‐centred theory: the conceptual and evidential rationale for a person‐centred approach to clinical practice. The role of person‐centred theory was highlighted in explanations for both how educational interventions failed and how they succeeded in developing person‐centredness. Previous studies have shown that a knowledge hierarchy in medical education, where objective facts are more highly valued, undermines the development of person‐centredness in learners.^100^, ^101^ Learners' values and professional role understanding has been shown to guide their engagement with learning, with more biomedical perspectives associated with poorer engagement with person‐centred learning.^102^, ^103^ It has also been observed that person‐centred skills training can lead to an increase in clinicians using certain behaviours without a change in their attitudes.^47^, ^104^ Our findings extend the learning from these studies by explaining how a lack of person‐centred theory in learning creates dissonance with existing perspectives on knowledge and role which either results in learners minimising the importance of such learning or incorporating learning into existing meaning perspectives. Our findings are in line with constructivist learning theories, which explain how learners construct new knowledge on the foundations of existing knowledge.^105^

Our results also show how person‐centred theory is relevant to an understanding of professional conduct. Previous studies have described medical students and doctors using emotional detachment as a strategy to avoid distress.^106^, ^107^ Our results showed that interventions that do not offer applied person‐centred theory may lead to dissonance with a biomedical understanding of role (disease management) and professionalism (emotional detachment) and thus to an avoidance of situations where learners do not perceive a clear biomedical role. Our findings also explain how applied person‐centred theory can help learners embrace their role in supporting patients in situations where their biomedical expertise is of limited use, by helping them develop an expanded understanding of professional role.

A recent study of 16 medical schools curricula in Canada questioned if learners are receiving teaching on person‐centred concepts.^108^ A lack of explicit person‐centred theory in medical education may help to explain why studies have shown that both students and doctors hold superficial and unclear understandings of person‐centredness.^109^, ^110^ Learners may regard person‐centred practice as ‘implicit’ and ‘obvious’^109^ in contrast to a paradigmatic shift in practice described in academic and policy papers.^15^, ^16^, ^17^, ^20^, ^27^, ^111^, ^112^ Superficial understandings of person‐centredness may explain the gap observed between clinicians' belief that they are person‐centred and their clinical practice.^110^ Furthermore, studies that use validated scales to attempt to measure learners' person‐centredness consistently show higher scores in ‘caring’ compared to the ‘sharing’.^4^, ^24^ It may be that ‘caring’ is more easily integrated into a biomedical perspective where one still sees one's role as ‘doing for’, whereas ‘sharing’ involves a shift in perspective to ‘working with’ which requires a facilitative, partnership approach with patients.^113^, ^114^ Without an explicit conceptual framework and evidential rationale for the theory of person‐centred practice,^21^ it may hard for learners to commit to person‐centred professional practice.

Our results showed that when person‐centred theory was applied to meaningful experiences, this triggered an understanding of the relevance of person‐centredness to clinical practice. Meaningful experiences included engaging with real patient stories, opportunities to get to know patients as people over time, as well as opportunities to take an active person‐centred role in their care. Our findings fit with a recent realist review that showed that access to patients' whole illness trajectories was an important context for learning about patient‐centredness.^101^ Our findings also support literature that calls for an increase in patient‐educators^115^ and active opportunities to care for patients.^116^ Our results help to extend an understanding of the literature on LICs, an educational approach that has been demonstrated to enhance person‐centredness,^11^, ^12^ by highlighting how these clinical placements may act as a resource for meaningful experiences.

### 4.3 Support for sense making

The opportunity for learners to process their emotions and critically reflect on their responses to learning, including dissonance, allowed them to develop new meaning frameworks and self‐awareness. Dobie has argued that learners can miss the fact that self‐awareness and self‐knowledge are crucial to person‐centred practice.^117^ She has called for education that supports the emotional work and critical reflection needed to develop self‐awareness to be the foundation for medical educational curricular reform. More recently, incorporation of dialogic learning into medical education has been proposed to enhance person‐centred practice.^118^ Dialogic learning involves regular opportunities for teachers and learners to meet in non‐hierarchical settings to reflect on patient experiences. In the wider health care context, Schwartz rounds, which provide multidisciplinary teams the opportunity to process their emotional and cognitive responses to clinical practice, have been shown to support person‐centred attitudes.^119^

The characteristics of interventions most likely to support sense making were regular small groups with relational continuity of learners and group facilitator. This continuity allowed relationships of trust to develop which enabled participants to feel safe enough to express their emotions, engage in honest appraisal and challenge their assumptions. These conditions mirror those known to foster transformative learning, which adds explanatory power to our findings.^120^ Transformative learning theory states that for adult learners to change their existing meaning framework, they need support to critically reflect on their values, meanings and purposes.^121^ In our review, we found that critical reflection and emotional processing took place together and this is supported by more recent work on fostering transformative learning which shows that the capacity for critical reflection may depend on the ability to process emotions.^122^

### 4.4 Relationship of educational interventions to whole curriculum

Our review did not find any person‐centred interventions that were perceived to be in line with the wider focus of the curriculum. All interventions, including those that applied theory to meaningful experiences and support for sense making, were perceived to be at odds with the biomedical focus of the curriculum and led some learners to minimise the importance of the learning for their future clinical practice. Our recognition of the importance of epistemological dissonance reinforces calls for a whole curriculum approach to support person‐centred practice.^123^

### 4.5 Strengths and limitations

By focusing our review on person‐centredness, which sits at the level of participant perspective, informed by values, attitudes and beliefs, we have been able to help address the gap in understanding around why medical education has not led to an increase in person‐centeredness and crucially what can be done about this. By using a realist approach, we have been able to infer the mechanisms that are triggered by the interaction of interventional components with learner perspectives. By choosing to focus our review on the planned curriculum, our review offers policymakers pragmatic findings that can be used to develop and test interventions to support the development of person‐centred doctors.

There are several limitations to our research findings. Apart from a few interventions,^40^, ^53^, ^58^, ^91^ most papers offered data on short‐term changes in participant perspectives. This review identifies a gap in the literature supporting evidence about longer term transformation towards a person‐centred perspective and how to support this. Our research focused on planned interventions that aimed to develop person‐centredness and did not include wider educational practices, such as assessment, which may impact the development of person‐centredness. Also, our research did interrogate how learner perspectives were formed, and future work is needed to examine the influence of health care delivery on the normalisation and practice of a person‐centred approach. Finally, a significant proportion of medical education takes place in the wider context of health care, and planned medical education has a limited influence in shaping this context. Therefore, our findings are only a partial answer to the larger question of how to support the development of person‐centred doctors.

### 4.6 Implications for educational practice

Our findings explain why a skills‐based approach may be insufficient to support the development of person‐centredness in doctors. In line with constructivist learning theory, our review finds that educational interventions interact with learners' existing meaning perspectives. Therefore, in order shift from a biomedical to a more person‐centred approach to clinical practice, medical students and doctors need to understand why person‐centred practice matters to health. This may be achieved by integrating explicit learning on the theory of person‐centeredness with opportunities to experience its relevance in clinical practice through meaningful experiences.

Educational approaches also need to recognise and address the emotional work needed for perspective transformation. Regular opportunities to process emotions and critically reflect on responses support the creation of new meaning frameworks that enable a shift in perspective towards person‐centredness. Regular, supportive small group learning, with continuity of peers and facilitators, needs to be integrated throughout the curriculum. Finally, to optimise the development of person‐centredness, these components need to be integrated at both pedagogical and whole curricular level.

### 4.7 Implications for research

Our realist review provides insights into evidence‐based strategies that may be effective in medical education settings, and our refined programme theory offers a testable theory for medical educators and policymakers to implement in practice. Further research should test and refine our theory through empirical work to develop and evaluate educational models in practice. In particular, we call for evaluation of whether implementation of our programme theory can lead to persistent person‐centredness in practice. Understanding the relevance of person‐centredness to practice is closely aligned with the concept of autonomous motivation in self‐determination theory.^124^ Autonomous motivation develops when learners understand the value of an activity and it aligns with their sense of self, and several empirical studies have shown that autonomous motivation is highly related to persistence of an activity.^125^, ^126^ Longitudinal studies to test if applied person‐centred theory can support a sustained change towards person‐centred approaches would be a valuable area for future research. Furthermore, although the focus of our review was on medical students and doctors, our findings may be relevant to other health professions.

## 5 CONCLUSION

Our findings offer explanations as to why communication skills‐based educational strategies may be insufficient to develop person‐centredness. Integrating person‐centred experiences with theory on why person‐centredness matters, and enabling support for sense making, may support perspective transformation towards person‐centredness. Our findings offer programme and policymakers testable theory to inform the development of medical education strategies that aim to support person‐centredness.

## CONFLICT OF INTEREST

None.

## AUTHOR CONTRIBUTIONS

AB was the NIHR research fellow on the project. AB led all stages of the study, carrying out the article screening and selection, coding, data analysis and synthesis and developing the programme theory. JR was the primary supervisor supporting the design of the study and subsequent analysis. JR and CM supported the application for NIHR funding. SG was the information specialist on the study and designed the search strategy and sourced the articles. KS was the methodological advisor on the study. All authors contributed to protocol development, and all made substantial contributions to screening, analysis and developing the programme theory. AB wrote the first draft of the manuscript that was then critically revised by JR, CM, SG, KS and SP. All authors approved the final version for publication and agree to be accountable for all aspects of the work.

## ETHICS STATEMENT

Ethical approval was not required for this review.

## Supporting information


**Appendix S1** Supporting Information

**Appendix S2** Supporting Information

**Appendix S3** Supporting Information
