# Supplementary material for: Optimising planned medical education strategies to develop learners' person‐centredness: A realist review
Source: Med Educ. 2021 Dec 22;56(5):489–503. doi: 10.1111/medu.14707 (PMC9306905; doi:10.1111/medu.14707)
Supplement: Supplementary file 1 — Appendix S1 Supporting Information [file MEDU-56-489-s002.docx]

**Appendix A: Initial Programme Theory**

**Realist Review question:** How, why, for whom, and in what circumstances do educational interventions that aim to promote a person-centred approach change medical professional's perspective?

**Three draft candidate programme theories** developed through scoping searches (<https://osf.io/qnkfh/?view_only=d1d36e578dd449a78ec62b13e2efebc5>, tacit theories held by our research team and using concepts from substantive educational learning theory

**Draft Programme theory 1: Theory of PCC – cognitive learning**

If education supports an understanding of the conceptual underpinnings of person-centred approach (patient as a person, bio-psycho-social, therapeutic alliance, sharing power & responsibility, doctor as a person) and the goal of medicine as facilitating health and wellbeing (functional and meaningful) in terms of what the concepts mean and why they are important (e.g. knowledge of determinants of health, biomedical rationale for holistic approach, biases and assumptions,  and evidence of improved outcomes) this will result in a person centred perspective because of alignment of professional role with person-centred framework.

The mechanisms through which the outcomes may be achieved are permission (justification of personal values), perspective transformation through critical thinking and practical reasoning.

Communication skills training in the behaviours of person-centred care will not always lead to person-centredness due to a lack of alignment of behaviours with professional identity and role (which may be rooted in biomedical diagnosis and management).

Contextual variation (for whom):  There is variation in the worldview that doctors and medical students have in terms of health, illness, role, purpose as they commence undergraduate and postgraduate education. Those with a biomedical view are more likely to need explicit education in the theory of person-centred care to enable person-centred values, beliefs and attitudes.

Assessment focussed on biomedical framework and lack of epistemological clarity may hinder outcomes.

**Draft Programme theory 2: (transformative learning environments) – constructivist learning.**

Transformative learning environments provide the opportunities for supported critical reflection on experience which enable the meaningful integration of person-centred concepts with clinical practice and lead to person-centred values, attitudes and beliefs. Experience can include clinical encounters, stories (patient narrative, books, films).

Transformative learning environments:

- Encourage students to reflect on and share their feelings and thoughts
- Are holistically oriented and consider the whole student in the learning process
- Cultivate awareness of alternate ways of learning
- Promote a safe place for exploration through trust and care
- Facilitate relationships among students and others
- An instructor who models reflective thinking
- Help students question reality in ways that promote shifts in worldview

**Draft Programme theory 3: Clinical Placements - experiential** **learning**

If clinical placements support the development of relationships with patients, then this may lead to person-centred perspectives through for example exposure to the functional impact of illness of patient’s life, opportunity to learn what matters to patients and the value of relationships.

If clinical placements support feedback on practice through continuity with preceptor and this preceptor role-models person-centred practice, then this may lead to person-centredness.

If students are given clinical tools that support a person-centred approach (e.g., biopsychosocial history talking templates, narrative history) then this encourages person-centredness through integration of theory and practice (a holistic approach to health and illness, eliciting patients concerns, values and goals and experiencing the healing potential of listening and empathy).
