# Supplementary material for: Optimising planned medical education strategies to develop learners' person‐centredness: A realist review
Source: Med Educ. 2021 Dec 22;56(5):489–503. doi: 10.1111/medu.14707 (PMC9306905; doi:10.1111/medu.14707)
Supplement: Supplementary file 2 — Appendix S2 Supporting Information [file MEDU-56-489-s003.docx]

**Appendix B Main database search strategies**

**See OSF project for detail of Scoping search strategies**

| **Database** | **Search date** | **Results** |
| --- | --- | --- |
| **Ovid MEDLINE(R) ALL <1946 to July 12, 2019>** | **15/07/2019** | **2248** |
| **Embase 1996 to 2019 Week 28** | **15/07/2019** | **1777** |
| **HMIC Health Management Information Consortium 1979 to May 2019** | **15/07/2019** | **63** |
| **ERIC** |  | **129** |
|  |  |  |
|  |  |  |
|  |  |  |
|  |  |  |
| **Total results before duplicates removed** |  | **4217** |

**Date 15-07-2019**

**Description of reason for search:** Broad search for PCC and medical education in doctors/medical students

**Sources:**

Ovid MEDLINE(R) ALL 1946 to July 12, 2019

2248 results

| 1. Patient-Centered Care/ |  |
| --- | --- |
| 2. Holistic Health/ |  |
| 3. (whole adj person).ti,ab. |  |
| 4. (whole adj patient*).ti,ab. |  |
| 5. ((person* or patient*) adj (centered or centred or orient* or focus*)).ti. or ((person* or patient*) adj (centered or centred or orient* or focus*)).ab. /freq=2 |  |
| 6. (biopsychosocial* or patient?centeredness or patient?centredness).ti,ab,kw. |  |
| 7. or/1-6 [ set for PCC ] |  |
| 8. exp Education, Medical/ |  |
| 9. Students, Medical/ |  |
| 10. (educat* or teach* or learn* or train*).ti. or (educat* or teach* or learn* or train*).ab. /freq=2 |  |
| 11. Curriculum/ |  |
| 12. 10 or 11 [ broad education/training search ] |  |
| 13. (physician* or medical or medicine or doctor*).ti,ab. |  |
| 14. exp Physicians/ |  |
| 15. Physician-Patient Relations/ |  |
| 16. 13 or 14 or 15 [ physician focus ] |  |
| 17. 12 and 16 [ physician combined with education ] |  |
| 18. 8 or 9 or 17 [ MESH medical education or physician + education terms ] |  |
| 19. 7 and 18 [ PCC AND medication education ] |  |
| 20. limit 19 to (english language and yr="2000 -Current") |  |

**Date 15-07-2019**

**Description of reason for search:** Broad search for PCC and medical education in doctors/medical students

**Sources:**

OVID Embase <1996 to 2019 Week 28>2248 results

| 1. (whole adj person).ti,ab. |  |
| --- | --- |
| 2. (whole adj patient*).ti,ab. |  |
| 3. ((person* or patient*) adj (centered or centred or orient* or focus*)).ti. or ((person* or patient*) adj (centered or centred or orient* or focus*)).ab. /freq=2 |  |
| 4. (biopsychosocial* or patient?centeredness or patient?centredness).ti,ab,kw. |  |
| 5. or/1-4 |  |
| 6. *medical education/ |  |
| 7. (educat* or teach* or learn* or train*).ti. or (educat* or teach* or learn* or train*).ab. /freq=2 |  |
| 8. (physician* or medical or medicine or doctor*).ti,ab. |  |
| 9. *physician/ |  |
| 10. doctor patient relation/ |  |
| 11. 8 or 9 or 10 [doctor set] |  |
| 12. 7 and 11 |  |
| 13. 6 or 12 |  |
| 14. 5 and 13 |  |
| 15. limit 14 to (english language and yr="2000 -Current") |  |

**Date 15-07-2019**

**Description of reason for search:** Broad search for PCC and medical education in doctors/medical students

**Sources:**

OVID HMIC Health Management Information Consortium 1979 to May 2019 63 results

| 1. exp patient centred care/ |  |
| --- | --- |
| 2. (whole adj person).ti,ab. |  |
| 3. (whole adj patient*).ti,ab. |  |
| 4. ((person* or patient*) adj (centered or centred or orient* or focus*)).ti. or ((person* or patient*) adj (centered or centred or orient* or focus*)).ab. /freq=2 |  |
| 5. (biopsychosocial* or patient?centeredness or patient?centredness).ti,ab. |  |
| 6. or/1-5 |  |
| 7. exp Medical education/ |  |
| 8. (educat* or teach* or learn* or train*).ti. or (educat* or teach* or learn* or train*).ab. /freq=2 |  |
| 9. (physician* or medical or medicine or doctor*).ti,ab. |  |
| 10. exp medical staff/ |  |
| 11. 9 or 10 |  |
| 12. 8 and 11 |  |
| 13. 7 or 12 |  |
| 14. 6 and 13 |  |
| 15. limit 14 to (yr="2000 -Current" and english)  **Date 15-07-2019**  **Description of reason for search:** Broad search for PCC and medical education in doctors/medical students  **Sources:**  ERIC (Education Resource Information Center) via EBSCO  S1 TI ( (person* or patient*) n1 (centered or centred or orient* or focus*) ) OR AB ( (person* or patient*) n1 (centered or centred or orient* or focus*) )  S2 TI ( biopsychosocial* or patient?centeredness or patient?centredness ) OR AB ( biopsychosocial* or patient?centeredness or patient?centredness )  S3 DE "Medical Education"  S4 TI ( educat* or teach* or learn* or train* ) OR AB ( educat* or teach* or learn* or train* )  S5 DE "Physicians"  S6 TI ( physician* or medical or medicine or doctor* ) OR AB ( physician* or medical or medicine or doctor* )  S7 S5 OR S6  S8 S4 AND S7  S9 S3 OR S8  S10 S1 OR S2  S11 (S1 OR S2) AND (S9 AND S10)  S12 (S1 OR S2) AND (S9 AND S10)  S13 (S1 OR S2) AND (S9 AND S10) Limiters - Date Published: 20000101-20191231 |  |
