# Supplementary material for: Optimising planned medical education strategies to develop learners' person‐centredness: A realist review
Source: Med Educ. 2021 Dec 22;56(5):489–503. doi: 10.1111/medu.14707 (PMC9306905; doi:10.1111/medu.14707)
Supplement: Supplementary file 3 — Appendix S3 Supporting Information [file MEDU-56-489-s001.docx]

**Appendix C: Characteristics of included studies**

| Study | Contribution to CIMOc | Country | Participants | Paper type | Study methodology | Intervention characteristics (where applicable) | Summary of planned education strategies | Summary of Findings  (Explanations as they relate to the three draft programme theories - PG1, PG2, PG3 on our initial programme theory) |
| --- | --- | --- | --- | --- | --- | --- | --- | --- |
| Arntfield (2013) | CIMOc 6 CIMOc 7 CIMOc 8 CIMOc 9 | USA | UG. 12 final year medical students | qualitative | Three methods of data collection. Weekly open-ended surveys, focus group at end of intervention, open-ended questions 18 months later. | 4 weeks narrative elective involving readings of patient stories with small group discussion and written reflections | Theory + narrative experience + sense-making | Perspective on good communication shifted from an ability to explain and listen to a deeper form of listening which truly respected patient story, and goals. Some felt their existing person-centred perspective was validated by intervention. Strong perceived link between learning outcomes and pedagogical approach of small safe non-competitive learning environment which enabled them to express views, hear diverse viewpoints and reflect critically on their responses to learning. Recognition of importance of development of self-awareness as part of development of person-centred professional identity. (PG2: Transformational learning environment). Intervention was perceived to be at odds with wider curriculum and odds with perspective of majority of medical student peers. Felt important to explain relevance of learning to clinical role to increase status of learning and transform perspective of majority. (PG1: Theory of PCC) |
| Bansal (2015) | CIMOc 6 CIMOc 9 | UK | PG (8 IMGs) | qualitative | Participant reflections. | Whole day course on theory of person-centred care with an opportunity to critically reflect on assumptions, biases, and impact on patient care. Reflection on application of learning to real patient care in small groups 2-3 weeks later. | Theory + practice + sense-making (no continuity) | Understanding the concept of health as functional and individualised as well as the rationale for PCC in terms of positive outcomes, helped support change in practice without behavioural skills training. Participant comments suggest perspective change, through valuing new approach and understanding relevance. Exploration of culture as individually complex contextual and intersectional supported approach to respectful curiosity. (PG1: Theory of PCC)  Self-awareness exercises reflecting on culture and assumptions in group helped inter-cultural communication. (PG2: Transformative learning environment) |
| Barr (2015) | CIMOc 1 CIMOc 2 CIMOc 6 | Australia | UG. Medicine. (Last two years) 67 questionnaire (qual), 14 2 x focus groups, 8 semi-structured interviews | qualitative | written questionnaire, focus groups and interview | Weekly small group teaching with GP mentor and patient with long-term complex disease. | Theory + Experience and sense-making | In the context of students having established a biomedical understanding of a doctor's role, an educational intervention which disrupts this by focussing on students learning person-centred attitudes from patients with chronic and complex conditions through consultation skills teaching does not lead to person-centred attitudes and values for majority due to dissonance and conflict with existing identity. Teaching skills without theory leads to instrumental use of approach for biomedical purposes. (PG1: Theory of PCC)  Likely that small group with continuity of facilitator in safe space enabled a minority of students able to start developing true person-centredness through reflection on the importance of patient context, experience, and goals to their illness. (PG2: Transformative learning environment)  Clinical placements perceived as task-based and biomedical and at odd with learning (PG3: Clinical place |
| Beckman (2012)  Krasner (2009) | CIMOc 7 CIMOc 9 | USA | PG (20 GP trainees) | qualitative  quantitative | Semi-structured interviews  Description of intervention. Pre and post questionnaires on validated scales for Burnout, empathy,  mindfulness, mood states and personality | 70 primary care physicians volunteered to participate in a 52-hour continuing education course that consisted of 8 weekly sessions, a  silent retreat, and 10 monthly sessions. The course included mindfulness meditation, self-awareness exercises, narratives about meaningful clinical experiences, appreciative interviews, didactic material, and discussion. | Theory + experience + sense-making | Non-judgemental and supportive small group space facilitated deep reflection on experiences and professional identity. Experiencing being heard allowed participants to recognise ‘being heard’ as therapeutic. Mindfulness training facilitated openness, respectful curiosity, and attentiveness in patient encounters. Not just as a behaviour but as an approach. (PG2: Transformative learning environment) |
| Blickem (2007) | CIMOc 6 | UK | PG (CPD). Ward based 10 nurses, 2 OT, 1 physio | qualitative | Analysis of two recorded interprofessional workshops discussing authentic fictional patient narratives | 3-hour multiprofessional workshops for hospital ward teams. Participants were given individual narratives to read and discuss in small groups/pairs and then feedback their responses to the rest of the team. | Narrative Experience and sense-making | Evidence that healthcare professionals’ concept of role involved control (this is my job), and knowledge expertise. Feeling of threat when encountering patients or carers with knowledge. This was barrier to 'working with' instead of 'working for' (PG1: Theory of PCC)  Reading in pairs and small group setting over 3 hours allowed for critically reflective practice. Narratives facilitated reflection on difficult terrain around power, role, poor care from patient point of view. Fictionalised and yet authentic narrative as an educational stimulus may be useful in giving access to perspective where personal experience may be too 'close to home' for clear critical reflection. (PG2: Transformative learning environment) |
| Bombeke (2012) Bombeke (2011) | CIMOc 2 | Belgium | UG and PG. 67 UG. 85 medical students | qualitative quantitative | Interviews and focus groups Cohort study. Validated questionnaires on PCC attitudes administered pre and post clerkships. One group received CST and other didn't. | Communication skills training with role-play (not real patients) across all five years of training. Based on Cambridge-Calgary consultation model. | Comms skills | Bombeke 2012: CST training may generate negative attitudes to person-centredness. Hierarchy and conceptual division of knowledge – PCC seen as less important 'soft’ than 'hard facts' for clinical practice. Multiple reasons: Lack of explicit learning on relevance of patient centred communication, not integrated with clinical practice, not integrated with clinical reasoning, standardised approach feels artificial, not using real patients. Students still feel uncomfortable with psychosocial questioning despite CST training when entering clinical practice and revert to biomedical approach. Bombeke 2011: CST training reduced patient-centred attitudes.  (PG1: Theory of PCC)  CST training may generate negative attitudes to person-centredness. Not discussed how to use approach flexibly depending on illness and patient context, not allowing students to find personal style. Critical reflection important to help develop authentic individual student style. (PG2: Transformative learning environment)  Communication encountered in clinical practice far from 'ideal' CST training caused confusion - some discarded as not relevant. Experiential learning helped recycle learning in 7postgraduate practice. From discussion: active role and responsibility will support opportunity to implement patient-centred communication and learn what works. (PG3: Clinical placements) |
| Boudreau (2007) Boudreau (2014) Boudreau (Unpublished survey data) | CIMOc 1  CIMOc 4  CIMOc 5  CIMOc 6  CIMOc 7  CIMOc 8  CIMOc 9 | Canada | UG. Medical students UG. 24 Medical students, 3 teachers UG. 340 medical students | n/a qualitative mixed methods | (2007) Description of intervention.  (2014) Case study design over 4 years with focus groups of students and faculty. Unpublished survey data and focus groups across 4 year cohorts (2008 - 2011) | Physicianship curriculum integrated across all four years of learning based on person-centred approach: that healing is the doctor’s primary obligation. Explicit theory, clinical methods training and small group learning over four years to reflect on experience. | Theory + sense-making | Boudreau 2007: conceptual clarity on goal of medicine as healing is fundamental pre-requisite to PCC education and is the underpinning concept around which medical curriculum should be structured.  Boudreau 2014: Initially most students’ worldview not PCC - focussed on biomedical doctor’s role (facts and skills) as ‘doing’ ‘cure’ and ‘acute’ medicine. Lack of sessions' congruence with student understanding of purpose led to initial frustration with intervention.  Unpublished survey data: Changes in ideas on the 'ideal physician' towards more person-centred perspective. Students describe person-centred concepts of individualisation, patient autonomy, therapeutic relationship, therapy in listening, collaboration with patient and self-awareness. Descriptions demonstrate greater conceptual richness and clarity in students undertaking intervention compared to students prior to this. Theory accessible through 1. reflection on practice with patients in small groups and reflection on emotional experiences. 2. integration of concepts with clinical application example. (PG1: Theory of PCC)  Boudreau 2014: Longitudinal small group teaching with tutors and senior students to allow self-reflection on experience within the PCC value framework. Safe space for emotional processing.  Unpublished survey data: With time able to integrate PCC concepts and healer role into professional values through critical refection on clinical experiences in a safe space with support from group and facilitator. Reflection more spontaneous (not forced) in clinical clerkships. Possibly due to greater material for reflection (relevance, integration) and possibly due to familiarity with process (habits of mind). Characteristics of tutor: role-modelling PCC, encouraging critical thinking, being caring, listening, enjoys working with students and acts as guide and mentor. Safe environment to share, lack of formal assessment and discussion with group also considered important to learning experience. (PG2: Transformative learning environment)  Person-centred professional role 'healer' not valued or recognised in clinical placements by clinical supervisors. Lack of role-modelling of PCC approach and feedback on clinical practice from clinical supervisors was barrier to PCC professional development. (PG3: Clinical placements) |
| Buist (2018) | CIMOc 6 | Australia | UG. Medicine 76 responses | mixed methods | questionnaire with Likert scales and free text boxes for qualitative data | 90min tutorial in small groups of 4-6 patients at bedside in hospital. Taking a traditional patient history and then revisiting patient and directing to ask thoughtful questions to follow up patient responses. | Experience and sense-making | Traditional clinical history taking tool restricts role to diagnostician of disease and is barrier to PCC. This may lead to inappropriate checklist interaction with patients even when diagnosis has been made and irrelevant to current problem. Leads to deliberately ignoring patent cues and concerns as no place for this on template. Increasing patient exposure will not necessarily lead to a person-centred approach if students approach patients with this biomedical diagnostic perspective. Supporting students to use a different more-person-centred approach to information gathering resulted more than half of students saying they would be following up on patient cues and understand problem from patient perspective but there was still resistance to this approach as perceived to be unhelpful to pass exams. Small groups with feedback from tutor over 4 to 5 weeks. (PG3: Clinical placements) |
| Centeno (2017) | CIMOc 6 CIMOc 7 CIMOc 8 CIMOc 9 | Spain | UG (20 2^nd^ and 6^th^ year medical students) | mixed methods | Written reflection, questionnaire Likert and focus group of 8 students | 20 hours over 6 days. Art + Didactic/cases/stories + clinical | Theory + Narrative experience + sense-making | Using arts, hearing patient stories, and engaging in supported critical reflection enabled perspective transformation to person-centeredness. Clinical staff role modelling pcc approach helped. Potential refinement re interdisciplinary team to model different perspectives and interprofessional collaboration. Potential refinement re not seeing death/chronic illness as a failure but focus on patient. (PG2: Transformative learning environment) |
| Cohen (2000) | CIMOc 6 | USA | UG. Medicine. Number not stated | mixed methods | questionnaires and focus groups | Large group with patient stories and faculty. Occasional small group working. | Narrative experience and sense-making | Early medical intervention that aims to promote a holistic approach to health and uses real patient stories promotes learning of key concepts of Person-centred practice and relevance to clinical practice. No explicit didactic conceptual learning. Theory made relevant through exploration of patient experience. (PG1: Theory of PCC)  Small group learning with reflection on real cases helped students consider ethical, legal, clinical issues and integrate relevance in decision making. Narrative as educational stimulus most impactful and memorable when delivered by patient themselves but real cases discussed in small groups also impactful. Facilitation best supported by broad holistic practitioner who role-modelled pcc approach. Active participation with patient important. Patient story with interprofessional faculty enabled appreciation of role of other healthcare workers in patient care. (PG2: Transformative learning environment) |
| Dahm (2013) | CIMOc 4 | Canada | PG (7 IMGs) | qualitative | Observation of 14 role-play consultations | Communication skills training | Communication skills | Tokenistic use of empathy and difficulty establishing rapport observed. Mechanism thought to be lack of understanding of PCC role of doctors and value of this approach. (PG1: Theory of PCC) |
| Domenech (2011) | CIMOc 6 | Spain | PG (170 physiotherapy students) | quantitative | 3 validated questionnaires before and after | 2 x 3-hour interventions (biomedical and biopsychosocial) | Theory + experience + Sense-making | Small amount (2 x 3 hours) of conceptual support on biopsychosocial (BPS) theory and evidence of impact on health outcomes from research papers changed beliefs and attitudes and this was correlated with management intention. Learning that focussed on biomedical aspects, resulted in change from pre-intervention towards less person-centred attitudes. Direct effect of implicit theory of teaching and not just lack of holistic concept despite practice guidance having a PCC frame. (PG1: Theory of PCC)  Intervention involved integration of theory with practice - active discussion of cases with patients. Control intervention more didactic. (PG2: Transformative learning environment) |
| Donetto (2012) | CIMOc 4 CIMOc 5 CIMOc 2 | UK | UG. 30 Medical students, 11 educators | qualitative | Observation of 87 small group sessions and interviews | Communication skills training | Communication skills | Students’ rationale for using person-centred communication skills approach is instrumental (better diagnosis and compliance) leading to mechanistic use in consultation skills sessions (encouraged by OSCE assessment). Behavioural approach to communication skills teaching did not encourage examination of power dynamic and could exist within hierarchical doctor-centred frame rather than collaborative approach of sharing power. Some students rejected 'performance of empathy' as inauthentic but saw as necessary for exams. Student conceptual framework positivist and hierarchy of hard facts over soft interpretivist knowledge. This epistemological distinction was not examined or challenged by curriculum and often reproduced by focus on science. (PG1: Theory of PCC)  Pedagogical approaches are key. Pedagogy of discomfort - inviting educators and students to question underlying assumptions. Readings as prompts for discussion on power dynamics, epistemology of medical science (what is fact and how is it produced). (PG2: Transformative learning environment) |
| Eikeland (2014) | CIMOc 3  CIMOc 5 | Norway | UG. 11 Medical Students (third year) | qualitative | Semi-structured interviews. Content analysis | Communication skills training completed, and students interviewed in the first year of clinical contact with patients. | Communication skills | Students' understanding of their role inhibited empathy because it demanded emotional detachment, objectivity, and a biomedical focus. Emotions may cloud thinking. The paper suggests these ideas of role may come from hidden curriculum rather than what students come into medical school with. (PG3: Clinical placements) |
| Gallentine (2014) | CIMOc 6 | USA | UG. 15 Medical students | qualitative | two focus groups (9 male and 6 female) | Didactic instruction on health and quality of life, values-based patient-centred care followed by communication and motivational skills training and then follow up of patients at home. | Theory + skills + experience | Students thought motivational interviewing skills training changed perspective towards pcc role to share control, empower and “work with the patient, not at the patient.” Also, greater understanding of individual patient and how they define health. Skills training came after conceptual learning and evaluation did not ask about what changed perspective and why. (PG1: Theory of PCC)  Despite education that moved them towards person-centredness, students found this approach difficult to maintain on placements given their pre-existing biomedical training that they fell and returned to biomedical task orientation of other medical professionals and medical organisational structures. However greater awareness of what they and others were doing and whether it addressed patient needs or blamed patient as the problem. (Could this difficulty in translating to practice be due to lack of critical reflection) (PG3: Clinical placements) |
| Garrison (2011) Garrison (2010) MedEdPortal | CIMOc 6 | USA | UG. 44 Medical Student | qualitative description of intervention | Analysis of students written reflections Written qualitative comments on intervention. | Context of 4-week psychiatry placement. Orientation to concepts of narrative approach and narrative therapy through tutor, discussion of approaches and impact on patient, observation of patient video, observation of film clip, practicing taking narrative history from peer in Paris, analysis of written vignettes (self-directed). Students given guide narrative interview template. Narrative interview, discussion with preceptor, discussion with patient (Approx. 4 hours over 4 weeks), reflective writing (which focussed on therapeutic effects of narrative process and reflection on therapeutic potential of doctor), discussion in groups. | Theory + narrative experience + sense-making | Garrison (2011) Clear orientation to concepts underpinning narrative educational intervention. Reading of concepts, discussion, practice and reflection supported students to benefit from experience. Garrison 2010: exercises before patient interview explore the idea of the patient-doctor interaction having therapeutic and non-therapeutic elements. Concept of therapeutic involves hope, control and empowerment (through explanation or strengths).  (PG1: Theory of PCC)  Critical reflection of experience through writing and in groups supported development of insight into value of approach. (PG2: Transformative learning environment)  Clinical tools (narrative history taking) which require capturing patient experience and perspective on problem, facilitate person-centred communication such as active listening and support person-centredness through recognition of value of patient perspective in management and therapeutic value of being heard, opening up, relationship building, and patient's gaining insight into their problems. Clinical tools that focus on hope and strengths may facilitate person-centredness by clearly focussing on person rather than problem. Reflection on how traditional medical interview dehumanises both doctors and patient.  Variable outcomes of reflection on experience seemed to be related to quality of patient experience. Greater involvement and feedback from preceptor asked by some as improvement. (PG3: Clinical placements) |
| Ghosh (2017) | CIMOc 6 | USA | UG (60 3^rd^ Year Medical students) | qualitative | Analysis of student case presentations. | Following cancer patient for 1 year (longitudinal) | Longitudinal experience | Observing care longitudinally from the patient perspective can enable students to recognise the importance of psychosocial aspects of life on health and function and the importance of ascertaining patient perspective in medical management. (PG3: Clinical placements) |
| Gonzalo (2018) Gonzalo (2020) | CIMOc 4 CIMOc 5 CIMOc 6 | USA | UG. 229 Medical students UG. 710 Medical Students | qualitative  qualitative | Thematic analysis of written log entries from students (792 logs from 363 unique patients) and six in-depth semi-structured interviews.  Thematic analysis of end of session and end of course written evaluations. | Didactic course on health systems (>100 contact hours) including knowledge and concepts and then patient navigator role of 2-3 afternoons per months over 9 months in interdisciplinary teams with a mentor. Student role to help patients navigate care system, overcome barriers to care, health coaching, patient education and transition planning. | Theory + experience | Gonzalo 2018: Detailed learning on knowledge of wider determinants and health system (PG1: Theory of PCC)  Large and small group discussion of theory (PG2: Transformative learning environment)  Patient navigator role. Active meaningful role with IPT over time can support an understanding of wider determinants of health, need to understand patient context and confidence with communication as well as role of wider team.  (PG3: Clinical placements) |
| Gordon (2014) | CIMOc 7 CIMOc 8 CIMOc 9 | USA | UG. Medical students (pre-clinical) | mixed methods | Faculty questionnaire | 4–12-week course in mind-body skills. Small group. Safe non-judgemental space | Theory and small group | Evidential rationale given for how mind-body techniques support health. (PG1: Theory of PCC)  Safe small groups (non-judgemental) allowed students to develop self-awareness. (PG2: Transformative learning environment) |
| Guerrero (2003) | CIMOc 6 | USA | UG. 16 Medical students | quantitative | Pre and post intervention questionnaire. Quantitative. Paired t-tests | Brief 2-hour intervention using BPS case diagramming | Theory + narrative experience + sense-making | Theory of BPS and links between bio, psycho. social and cultural aspects of patient experience to core role of diagnostic formulation and management made clear through patient case. Even brief intervention improved confidence and helped students see importance in patient care. (PG1: Theory of PCC) |
| Hudson (2016) | CIMOc 1 | Australia | UG. 279 Medical students (first year graduate entry) | quantitative | Pre (at entry) and post intervention (7 months after) questionnaire. Readiness for Interprofessional Learning Scale including measure of patient-centredness (5 out of 26) | 3-week clinical placement immersion in teams with nurse or allied professional as preceptor. | Experience (clinical placement) | Under-developed professional identities more likely to be challenged by clinical exposure. (PG1: Theory of PCC)  Authors conclude that sense-making opportunities (in transformational spaces) that allow professional identity formation crucial for attitudinal change from clinical experience. (PG2: Transformative learning environment)  Clinical exposure to patients will not always lead to increased patient-centredness if lack of active participation, short in length so less continuity and lack of sense-making opportunity (PG3: Clinical placements) |
| Kjeldman (2003) | CIMOc 9 | Sweden | CPD. 41 experienced GPs | quantitative | questionnaire scale. T-tests and univariate analysis | Weekly of fortnightly small group (6-10) GP Balint groups with 1-2 expert facilitators over many years. Continuity of group and mentorship. Length of reflection not stated.? 1-2 hours. | Theory + experience + sense-making | Some support for holistic epistemology supporting person-centred attitudes towards patient problems. (PG1: Theory of PCC)  Critical reflection on patient encounters in real clinical practice in a safe space, with expert facilitation, supports the development of a person-centred attitude in terms of welcoming all patient problems, psychological etc. The longer the training (>1 year) and the frequency (1-2 weekly) the greater the impact. Critical reflection enabled better relationships, encouraged continuity of relationships with patients over time and improved sense of control over work, reduced stress (PG2: Transformative learning environment) |
| Krupat (2009) | CIMOc 6 CIMOc 7 | USA | UG. 49 Medical Students. First clinical year | quantitative | Completion of Patient-Practitioner Orientation Scale (PPOS) at beginning and end of year |  | Experience and sense-making | Knowledge taught in interdisciplinary case-based learning emphasising integration of basic sciences may have supported a more holistic understanding of illness relevant to practice as from real patient cases. (PG1: Theory of PCC)  Protected time for students to come together guided by a faculty preceptor to discuss their experiences and to process feelings about their experiences in the hospital and their ambulatory settings. Groups together for a year so student peer group support due to relationships through continuity. (PG2: Transformative learning environment)  Longitudinal experiences enabled students to form meaningful relationships with patients over time. (PG3: Clinical placements) |
| Kumagai (2009) | CIMOc 6, CIMOc 7 CIMOc 8 CIMOc 9 | USA | UG.12 Medical students pre-clinical | qualitative | individual interviews. Grounded theory. | Contact with diabetes patients in pairs over 2 years. Regular small group facilitation with trained faculty to make sense of experience. Continuity with small group peer and preceptor. | (Theory)+ longitudinal experience + sense-making | Giving pre-clinical medical students a combination of a powerful experiential stimulus (real longitudinal patient contact) that engages affect with the sense-making opportunity from critical reflection in group leads to a new person-centred framework of understanding of role (importance of understanding patient context, perspective and goals; Importance of individualisation of care; importance of sharing power through explanation, importance of partnership with patient).  Diversity of patient illness stories in small groups promoted understanding of individualisation of care (PG2: Transformative learning environment)  Learning enhanced by continuity with patient that engages affect, enables direct observation of illness experience and forms relationship (PG3: Clinical placements) |
| Lavoie (2013) | CIMOc 6 | Canada | PG. 51 Nurses, doctors, social workers, pharmacists, volunteers | qualitative | Pre and post intervention semi-structured interviews of participants and relatives for outcome data. Analysis of medical records | Intensive complex longitudinal intervention (12 months). Starts with theory then case studies and reflective practice from real-life cases. Interprofessional. Collaborative co-creation of tools. Availability of mentor weekly 3 hours for 4 months to help integrate theory to practice. | Theory + experience + sense-making | Perspective transformation: The main focus of care shifted from the task to the patient. From 'doing care' to understanding what unique care was needed from patient perspective and therefore care grounded in listening and presence (patient as a person). From being cordial to being a helping presence (therapeutic alliance). From 'leading caring' to 'adapting to patients' and therefore autonomy more important than patient safety - persuasion to proposal (sharing power). Greater interprofessional team appreciation. For minority of all professionals, there was no shift in perspective as already felt had PCC approach. (PG1: Theory of PCC)  Likely that reflective small groups supported this learning. (PG2: Transformative learning environment)  Co-created tools changed documentation. Feedback likely supported integration of theory and practice. Increased amount of documentation on patient values, perspectives and needs (PG3: Clinical placements) |
| Law (2011) | CIMOc 6 | UK | UG. Medical Students | qualitative | extraction of quotes from reflective assignments | 4-week SSC for second- and third-year students. Books and film (biography and fiction) used as educational stimulus. Small group reflection with prompt questions weekly | Theory + narrative experience + sense-making | Reflection on patient narrative through film/books may allow a greater understanding of the patient perspective. Critical reflection in small safe groups enabled challenge to their assumptions and biases. Reflection needs to be practiced in safe small group environment over time to become deep and critical. (PG2: Transformative learning environment) |
| Loh (2018) | CIMOc 6 | Singapore | UG. 105 Medical Students. 3^rd^ year | mixed methods | survey asking yes/no questions and free-text reflective comments | Small groups created a video during family medicine attachment linking medical condition with a patient narrative and then reflected on lessons learned. | Experience + sense-making | Processing of patient narrative through creative project in group may have stimulated reflection from disease to illness-based perspective. (PG 2: Transformative learning environment) |
| Lumma-Sellenthin (2009) | CIMOc 4 CIMOc 2 | Sweden | UG. Medical students | qualitative | Observation of videotaped small group discussions of student-patient interviews of two small groups over first two terms of five-term course | Students meet every two weeks in small groups with a GP in primary care to discuss videotaped consultations they have had with patients. Interventions lasts five terms. | Communication skills | Lack of understanding of PCC conceptually, lack of understanding how to use empathy and function of it prevented use of pcc approach to communication despite skills template (PG1: Theory of PCC) |
| Marcus (2011) | CIMOc 4 | South Africa | UG (1^st^ year medical and dental) | qualitative | Analysis of written reflections | Five community clinic visits | Experience (clinical placements) | Lack of grounding in social science theory leads to lack of integration of experiential learning. Knowledge understood as facts leads to reduced validity of interpretive knowledge. (PG1: Theory of PCC) |
| Margalit (2005) | CIMOc 6 CIMOc 7 | Israel | PG. 44 GP trainees | quantitative | Pre and post intervention questionnaire. Post 6 months after intervention. Clinical knowledge and BPS orientation scored by blinded GPs in response to free text responses to questions. Attitudes to PCC on Likert scaler. | 12 weeks of small group teaching lasting 4-6 hours with trained clinical facilitators | Theory + experience + sense-making | Pre-intervention Biopsychosocial model of practice very low (16/100) and post intervention high (76/100). Suggests perspective transformation. Intervention included short theoretical background aligned to PCC philosophy - problems seen holistically, relationship as healing, understanding own inner responses, enablement, collaborative approach. (PG1: Theory of PCC)  Majority of increase in biopsychosocial model of practice and person-centred intentions likely to have come from shared intervention element: discussion of real patients in small groups with critical reflection. Experienced facilitator supported participants to develop shared solutions from BPS perspective. Integration of theory and practice and biomedical and psychosocial aspects of problem and how this applied to problem presentation and management. Discussed their experiences and difficulties in implementation a week later (critical feedback and closing of experiential learning loop). Increased burnout score - may be increased awareness of task. (PG2: Transformative learning environment) |
| McCLain (2004) | CIMOc 6 | USA | PG. psychiatry trainees (33 year 1, 46 year 2) | quantitative | expert grading of portfolio entries re biopsychosocial diagnostic formulation | Trainees asked to write a BPS formulation of patient they encountered with instructions. In one institution department lead advised faculty and residents in groups and individually of value and importance of BPS formulation. Subsequently led to feedback at bedside and specific teaching sessions on BPS formulation. | Theory + experience | Intervention involved specific didactic teaching on BPS *model and its value* in clinical diagnostic practice by lead and other faculty. Leading to significant increase from gathering information but not integrating, to integrating information competently. (PG1: Theory of PCC)  Bedside teaching with feedback on practice improved ability to practice but without intervention as residents spent longer in practice, they declined in their holistic formulation suggesting the context of clinical placements erodes PCC. (PG3: Clinical placements) |
| Meitar (2009) | CIMOc 1 CIMOc 3 | Israel | UG. 103 Medical Students (sixth year) | qualitative | Analysis of students’ written reflections on their feeling about breaking bad news to hypothetical written patient cases following communication skills course to learn to break bad news. | Course involved SPIKES training (2 didactic lectures, 4-hour panel with patients telling stories of their experience of receiving bad news and 14 hours of small group communication skills training) | Narrative experience + Communication skills | These findings empirically demonstrate that intrapersonal difficulties within the communicator and his or her level of self-awareness about them influenced the manner and content of the communication during the encounter. Findings suggests that enhancing self-awareness and addressing personal and professional difficulties could help physicians’ capability to cope with challenging communication tasks (PG2: Transformative learning environments) |
| Nagano (2019) | CIMOc 6 | Japan | UG medical students and PG doctors in first 4 years post-qualification | qualitative | reflective journal based on two open questions around changes to practice and changes to understanding of doctor role. Short semi-structured interviews with same format. | Accompanying person-centred senior doctors on home visits to patients at end-of-life where the focus is on their quality of life (not cure) with opportunity to reflect on experience. | Experience and sense-making | Experience can help move students/young doctors’ attitudes towards person centred care through recognition of utility of understanding patients view, goals, context (including relationships) and seeing them as person not a condition. It can also help to reconceptualise role and purpose of medicine towards person-centred practice "act of pursuing happiness “, recognition of multiple influences of health outside of healthcare. Reflections were not in depth and may show perspective transformation as starting from a very biomedical perspective. (PG3: Clinical placements) |
| Noble (+contact with author) (2007) | CIMOc 6 | UK | UG. Medical students. First 2 years (199 and 255) | quantitative | pre and post intervention questionnaire at start of year 1 and end of year 2. (48 item Doctor-patient validated questionnaire for person-centred attitudes) | Comparison of old curriculum (Y2 comm skills through lecture, role-play and one visit to patient. Essay question) vs new curriculum (15 sessions of communication skills over 2 years – lecture/video presentations and small group work. Greater contact with patients and talks from carer groups, ethics focus as well as half-day weekly small group teaching with same tutor integrating communication, clinical skills, ethics, health promotion etc. Patient scenarios used as platform for discussion and Reflection. Formative assessment. | Theory + experience + sense-making | Cohort with gains in patient-centred attitudes had learning sessions with an explicit focus on the training for the role of the doctor which integrates learning from science, social science, ethics, communication etc as basis of weekly small group sessions. Also received an explanation of the concept of patient-centred care is (based on the biopsychosocial model) (PG1: Theory of PCC)  Weekly small group learning with same tutor over two years. using patient vignettes as a springboard for discussion and reflection on a diverse range of integrated subjects with an emphasis on relevance to practice. Reflection on communication skills. Increased exposure to real patients and carers and hearing of their experiences may have increase appreciation of patient perspective. Communication skills training only without critical reflection did not lead to increase person-centredness and led to increased nervousness with interacting with patients. (PG2: Transformative learning environment) |
| Norfolk (+contact with author) (2009) | CIMOc 6 | UK | PG. 47 GP trainees | quantitative | questionnaires to assess attitude, knowledge, motivation and confidence towards therapeutic rapport at 4 points in three stage intervention as well as objective assessment of therapeutic rapport skills in videoed consultations | Full day of training (theory, reflection on videos and role-play with feedback) – 3 months of reflective log in practice and ½ day revisiting principles and small group reflection | Theory + experience+ sense-making | Explicit grounding of intervention in conceptual model of therapeutic rapport with emphasis on how important it is to feel motivated to be interested in patient perspective. Empathic motivation developed through rational utility (how it helps elicit useful diagnostic information, better relationships, better outcomes for all) and through how supports communication and shared understanding. Also, through self-reflection exercise of an empathic person they know and impact of that on them. Strong evidence for intervention being reason for increase in attitudes and behaviours (given no change in control group and observable change in behaviours) (PG1: Theory of PCC)  Experiential learning after conceptual grounding in small groups. Small group analysis of videoed consultations and participating and analysing small group consultations. F2F training had more impact that written reflective logs in terms of attitude and motivation. (PG2: Transformative learning environment) |
| Onyekere (2016) | CIMOc 6 | USA | UG. 31 Medical students. 2^nd^ year | qualitative | Analysis of video-recorded reflection sessions which were part of programme. | 2-day orientation to theory with communication skills training. Volunteering with MDT teams over whole academic year – working with patients with complex ‘non-medical’ needs. | Theory + experience | Initial brief theory - 2 days of lectures, role play and discussion including principles of PCMH and motivational interviewing. In weekly reflection sessions some knowledge content: Medicaid expansion, community services, and motivational interviewing. (PG1: Theory of PCC)  Working with MDT weekly for 8-9 months. Assessing patient needs, discussing with team, researching support. Working collaboratively with the patient and the PCMH team. Students were encouraged to develop longitudinal relationships and address multiple needs with their patients. (PG3: Clinical placements) |
| Puvanendran (2012) | CIMOc 6 | Singapore | UG. 44 Medical students. Third year | qualitative | Thematic analysis of three reflective essays at three time points. | Following two patients identified in hospital over 10months in home and at clinical visits. Limited small group reflection x2 | Experience | Very few opportunities for small group discussion. Reflective writing underdeveloped in students who were not educated to reflect. (PG2: Transformative learning environment)  Longitudinal experience with patients (over 10 months) increased person-centredness through providing appropriate contexts to learn the importance of this approach. The setting of home, community and chronic disease (cure and diagnosis not relevant) support person-centredness (patient context, perspective, goals, patient self-management, patient education). Powerful quote on how seeing same patient in hospital and then later at home supported reflection on purpose of care for patient. Reflections in hospital setting were largely biomedical despite suggestion of reflection on person-centred aspects. (PG3: Clinical placements) |
| Ratanawongsa (2009)  Ratanawongsa (2011) | CIMOc 6 CIMOc 9 | USA | PG. Doctors (first three years) PG. 40 Doctors (first three years) | qualitative (reflections, not systematic) quantitative | Quotes of experiences. No systematic qualitative analysis.  Online post intervention questionnaire with Likert scales. Comparison made of intervention with standard rotations | All interns and year 2 and 3 residents have one attachment on the Aliki team with fewer patients to admit, more feedback and reflection.   Aliki team: Admit half the usual patients during short- and long-call: time for knowing patients as individuals, improving patient transitions of care, and reducing barriers to medication adherence. Attendings observe and give feedback to residents in required activities: post discharge telephone calls to all patients, home visits to selected patients, telephone calls with outpatient providers, and structured interviews about medications. | (Theory) + Experience + sense-making | Monthly critical reflection facilitated in small group teams helped doctors to reflection on the holistic nature of health and helped them challenge their assumptions about patients.  (PG2: Transformative learning environment)  Experience of active caring for patients with person-centred focus supported importance of understanding individualised approach. (PG3: Clinical placements) |
| Ross (2011) | CIMOc 6, CIMOc 7, CIMOc 8 CIMOc 9 | USA | UG. 49 Physical therapy students | quantitative | validated survey instruments and open-ended written questions | Explicit course on person-centred framework (32 contact hours) including diversity, self-reflection, narrative and reflection in group. The concept of mindful practice was incorporated into each session | Theory + narrative experience + sense-making | Statistical difference in attitudes after conceptual course towards person-centredness. patient as a person, patient perspective, healing through relationship, self-awareness. Qualitative comments and consistency of score pre-interventions strongly support change due to intervention. Sharing element of PPOS affected more than caring. Perspective change around partnership and collaboration concepts. Caring may be able to exist within doctor-centred approach but sharing requires partnership approach (PG1: Theory of PCC)  Large group with continuity, facilitated discussion and reflection over several weeks (PG2: Transformative learning environment) |
| Satterfield (2004) | CIMOc 4 | USA | UG. Medical students. First two years | qualitative (few reflections – not systematic) | Student town hall and focus groups. | Didactic case-based integration of social and behavioural sciences across all pre-clinical learning with social science, science and clinical faculty | Theory and sense-making | Didactic education on social and behavioural sciences in pre-clinical context when students have limited patient contact may be rejected as unnecessary or 'common sense' by some medical students who have a biomedical perspective on role (if I learn the facts i can do the job). On the other hand, some students may be enthusiastic as this fits with their existing conception of the role of medicine. Therefore, the course may not lead to perspective change but may give some students 'permission' for their worldview. (PG1: Theory of PCC)  Conceptual learning without critical reflection to allow sense-making may lead to rejection/non-integration of knowledge. (PG2: Transformative learning environment)  Active responsibility and real patient contact may be needed to make conceptual learning relevant. (PG3: Clinical placements) |
| Saunders (2007) | CIMOc 7 CIMOc 9 | USA | UG. 82 Medical students (pre-clinical) | qualitative | Qualitative analysis of free-text responses to questionnaire | 11 weeks elective course of mind-body skills. 2 hours per week. Continuity of group and tutor. Complex intervention. Knowledge of scientific basis, experience of mind-body techniques, opportunity to reflect through writing, drawing, group. | Theory and sense-making | Evidential rationale given for how mind-body techniques support health. (PG1: Theory of PCC)  Continuity over 11 weeks with two tutors and 8-12 students in safe care non-judgemental space with opportunity for self-reflection, especially on emotions (PG2: Transformative learning environment) |
| Scavenius (2006) | CIMOc 6 | Netherlands | UG (489 1^st^ year) | qualitative | Analysis of case presentations. | 4 weeks working as assistant nurses in a hospital or nursing home at the end of their ﬁrst year of study. Students are on their own during these weeks of practical work; they are not given faculty guidance or coaching. | Experience | Being explicitly responsible for the wellbeing of their patient and primacy of patient autonomy, exposure to the caring role as opposed to clinical decision making and or early responsibility for caring in training (i.e., capitalising on idealistic tendencies). Also, refinement concerning the length of time in the placement, getting to know the individual, and experience of being lower in the hierarchy. (PG3: Clinical placements) |
| Schei (2019) | CIMOc 3 | Norway | UG. Medical Students (28 first year) | qualitative | Descriptive analysis of written reflections of random sample of 28 students | 6-month long mandatory course where first-year medical students (in groups of 4) do an unaccompanied home visit to a seriously ill patient. Patient and student group subsequently convene in class and share stories and impressions with 80 peers in an interactive plenary session, with 2 physician-instructors. In total, students participate in ten 3-hour sessions, each comprising 2 patient presentations and 1 reflective session exploring aspects of sickness, personhood, physicianship, and patient-centered medicine. | Experience | Experience with unwell patient was tricky for students with underdeveloped professional identity who were at a loss of how to manage these encounters. Uncertainty about professionalism and role. (PG3: Clinical placements) |
| Sturmberg (2005) Sturmberg (2002) | CIMOc 5 | Australia | UG and PG (GPs) UG. 12 4^th^ year medical students | n/a qualitative data | Description of intervention. No outcomes.  Course organisers reflections. Noted comparative examination performance of intervention and control group | BPSS approach 12 months in rural health placement following patients, weekly integration tutorial. Focus on symptoms | Theory and longitudinal experience | Sturmberg 2005: Concept of health as a balance within the BPSS framework. This framework is a tool to help students connect how all domains contribute to health and illness and recognise their role in addressing health in a holistic way. (PG1: Theory of PCC)  Weekly one to one opportunity to reflect on patients’ cases to integrate BPSS model of health. Clinical reasoning approach to history taking to help put learning into context. (PG2: Transformative learning environment)  Sturmberg 2002: Perspective transformation was not easy: Students who has already had 3 years of disease-focussed learning struggled to shift paradigm to person-centred approach and were concerned about impact of patient- based learning on assessment which was based on knowledge. A lack of explanation of philosophy led to discomfort and resistance from tutors (PG1: Theory of PCC)  Following patients in different contexts along with weekly tutorials for feedback and integration of holistic health dimensions was appropriate pedagogical approach to understand relevance of person-centred approach but lack of pcc orientation led to less than full uptake of opportunities. (PG3: Clinical placements) |
| Sweeney (2018) | CIMOc 6  CIMOc 8 | UK | UG. 48 Medical Students (third year) | quantitative | PPOS scale administered pre and post a 90 - minute session. (81% response to PPOS. Written feedback (73% response - not analysed) | Students shown three videos of patient talking about their illness experience and then a facilitated discussion of their reflections on the videos in small groups of 6-8 students. | Narrative experience + sense-making | sense-making in small groups for 90minutes. No continuity of group but there was a link to greater understanding through recognising a diversity of perspectives. (PG2: Transformative learning environment) |
| Tait (2013) | CIMOc 5 CIMOc 1 CIMOc 3 | Canada | PG 7 GP and 5 psychiatry trainees | qualitative | Interviews. 7 family medicine and 5 psych residents | Participants used a 1 hour “dignity interview” with dying patients | Experience | Participants reflected on their undergraduate curriculum in response to intervention. Stated that developing person-centred communication required meaningful learning with real patients and opportunity to critically reflect on experience as skills training along algorithmic. (PG2: Transformative learning environment)  Reflections on how dignity interview contrasted with existing understanding of professional role in biomedical ‘diagnoses and ‘cure’ terms and professionalism as maintaining an emotional distance. Supported understanding of subjectively defined goals, therapeutic function of empathy and attention, self-awareness. Lack of role-modelling and feedback on difficult experiences on clinical placements hindered learning. (PG3: Clinical placements) |
| Toivonen (2017) | CIMOc 1  CIMOc 3 | Finland | UG. 351 Medical Students (End of Fourth Year) | qualitative | Students do a written reflection on a patient case involving breaking bad news. | Students do communications skills training with actors in small groups with reflection over 1 year. They also have 2 hours introductory lectures. | Communication skills | Despite learning skills of how to break bad news, students felt tension between their role as professionals which they believed required emotional detachment and being able to empathise. There was also a feeling of uncertainty and helplessness due to feeling a loss of role (not being able to help through cure). The skills training had not helped this dilemma and not enabled them to connect with patients who are suffering. Some students talked of privilege of accompanying patient in their journey. (PG 1: Theory of PCC) |
| Tsimtsiou (2017) | CIMOc 6 CIMOc 8 | Greece | PG. 14 Doctors | mixed methods | Evaluation questionnaire with Likert scale and thematic content analysis of open-ended questions | 14 dermatologists randomly asked to participate in a course on non-technical skills: doctor-patient relationship consisting of 7-9 one to one coaching sessions lasting one hour. Applied theory in didactic sessions with examples from practice and videos to illustrate theory. Theory and evidence for person-centred approach as principles of communication, NLP, motivational interviewing, and self-care. | Theory + experience + sense-making | Applied theory changed understanding or role and importance of patient-physician relationship and practice moved towards person-centredness. (PG1: Theory of PCC)  Variation in responses to one-to-one sense making. Some liked this flexible approach that was individualised to them, but many spoke that having small group learning would have supported breadth and depth of learning from both an exposure to a variety of experience and different reflections on learning. (PG2: Transformative learning environment) |
| Van Es (2012) | CIMOc 2 | Netherlands | PG. 68 first year GP trainees | quantitative | t-tests and correlational analysis to understand relationship between MG score from OSCE type consultations and trainee personal characteristics | GP trainees has variation in communication skills training. Those from Netherlands had extensive training and IMGs had less. | Communication skills | Communication skills training does not lead to greater person-centredness. Assessed through an OSCE. (PG3: Theory of PCC) |
| Wahlqvist (2005) | CIMOc 2 CIMOc 3 | Sweden | UG. 12 Medical Students (final year) | qualitative | Video recordings of consultations from GP placement in final year. Recordings independently analysed by four educators with long experience of consultations skills training in UG and PG medicine. Then detailed discussion in 3-hr focus group interview 1 week after viewing recordings. | Students taught consultation skills over 9 weeks placement. | Communication skills | Skills training does not lead to greater person-centredness. Students have instrumental approach to tasks and remain doctor centred. Students ask questions and given time for patient story without really being interested in patient, without picking up on cues or an individualised shared approach into management plan. (PG1: Theory of PCC) |
| Warwick (2014) | CIMOc 4 | UK | PG. 12 GP trainees | qualitative analysis. | 2 focus groups of six doctors of 90 minutes each. | Communication skills training using person-centred approach with exploration of ICE without person-centred theory | Communication skills | The concepts of person-centred care and ‘reflection as an importance process in learning’ was new to International Medical graduates and far removed from their biomedical perspective as a result of acute disease-based education and experience. They felt the need to understand what was meant by being person-centred and its relevance to clinical care to be able to use ‘ideas, concerns, and expectations’ in communication skills training. (PG1: Theory of PCC) |
| Wilcox (2018) | CIMOc 5 CIMOc 6 | USA | UG. 156/498 Medical Students. | mixed methods | Survey of students with a modified Communication, Curriculum and Culture instrument with Likert scales and space for qualitative responses (thematic analysis). | Year 1 and 2 longitudinal experiences of 17-22 weeks. Year 3 and 4 traditional hospital blocks. | Longitudinal experience in first two years | Increasing patient exposure in clinical years corresponded to decreased person-centredness. Likely due to type of exposure. Hospital-based exposure likely to discourage person-centredness with a biomedical focus. Focus on explicit curricula (as in longitudinal curriculum in Y1 & Y2) and attitudes and behaviours of senior clinicians important. (PG3: Clinical Placements) |
| Yamada (2003) | CIMOc 6 | USA (Hawaii - multicultural) | UG (260 3rd year) | qualitative | Written essay (260). | Four-session seminar on the patient-physician relationship. Writing Assignments: Students are instructed to portray their family dynamics, their families’ beliefs about illness, note the influence of those beliefs on the course of the illness or treatment, and delineate the influence of their families’ values on their views of the practice of medicine. The students then present their narratives in a small-group session of family practice clerks, led by the authors | Theory, + experience + sense-making  (No sg continuity) | Self-reflection on family illness increased understanding of the many influences on experience of illness (cultural, family dynamic etc). Increased commitment to broader inquiry e.g., considering family. Also, reflection on holistic needs for compassion, hope, listening, validation. (PG3: Clinical placements) |
